# Chemotherapy-Induced Mucositis in Pediatric Oncology: Experience From 145 Cases at the Mohammed VI University Hospital, Oujda

**DOI:** 10.7759/cureus.82885

**Published:** 2025-04-24

**Authors:** Hanae Bahari, Ayad Ghanam, Hanane Hajaj, Aziza Elouali, Abdeladim Babakhouya, Maria Rkain

**Affiliations:** 1 Department of Pediatrics, Mohammed VI University Hospital, Oujda, MAR; 2 Faculty of Medicine and Pharmacy, Mohammed First University, Oujda, MAR

**Keywords:** chemotherapy, lips, oral mucositis, pediatric cancer, stomatitis

## Abstract

Introduction

Chemotherapy-induced mucositis is a common and debilitating complication in pediatric oncology patients, characterized by painful inflammation of the oral and gastrointestinal mucosal membranes. It commonly contributes to a decline in the quality of life, an elevated risk of infections, and prolonged durations of hospitalization. Management involves both preventive and therapeutic measures intended to reduce symptoms and prevent the onset of complications.

This study aims to evaluate the clinical characteristics, associated risk factors, and the effectiveness of therapeutic strategies in managing chemotherapy-induced mucositis in children treated at the Pediatric Oncology Unit of Mohammed VI University Hospital in Oujda.

Materials and methods

This retrospective study was conducted over 14 months, from July 2022 to August 2023, at the Pediatric Oncology Unit of Centre Hospitalier Universitaire Mohammed VI, Oujda. One hundred and forty-five pediatric patients receiving chemotherapy were included in the study. Data were systematically collected and recorded in an Excel file to document relevant clinical, demographic, laboratory, and treatment information.

Results

The age of the patients ranged from three to 16 years, with a male predominance of 66% (96 cases). In 51% of the patients (74 cases), a history of oral mucositis episodes was reported. Common hematologic malignancies observed were leukemia (41.3%; 60 cases), followed by lymphoma (20.6%; 30 cases), neuroblastoma (13.7%; 20 cases), bone tumor (10.3%; 15 cases), retinoblastoma (7.6%; 11 cases) and other tumors (6.2%; 9 cases). Psychological distress, including anxiety, was present in 66% of patients. Almost all patients exhibited anorexia and refused to eat. The intensity of pain, evaluated using the Visual Analog Scale (VAS), ranged from 4 to 9/10, necessitating opioid analgesia in certain cases. Laboratory findings revealed neutropenia in 80% (116 cases), lymphopenia in 33% (48 cases), and an elevated C-reactive protein (CRP) in 79% (114 cases) of the patients. The treatment approach involved the administration of topical antifungal agents and meticulous oral hygiene, which included the use of bicarbonate mouthwashes. In severe cases, intravenous antibiotic therapy was initiated along with multimodal analgesic management.

Conclusion

Oral mucositis is a frequent and severe side effect of chemotherapy, associated with considerable morbidity and a decline in quality of life, underscoring the importance of prompt diagnosis and intervention.

## Introduction

Oral mucositis (OM) is a prevalent adverse effect of chemotherapy in pediatric oncology, resulting from the cytotoxicity of antineoplastic agents on the rapidly proliferating epithelial cells of the gastrointestinal mucosa. This inflammatory condition is clinically characterized by erythema and ulcerations in the oral cavity, and is often associated with severe pain, impaired oral intake, and increased susceptibility to infections. These complications may necessitate intensive analgesic management, extend hospital stays, and compromise treatment continuity through chemotherapy delays or dose reductions, ultimately influencing therapeutic outcomes and quality of life [1,2].

The reported incidence of OM in pediatric patients with cancer varies widely, ranging from 16.7% to 91.5% [3], with some studies documenting rates between 20% and 80% [4]. Its occurrence is modulated by factors such as the cancer type, treatment protocol, age, oral hygiene, and immunological status, particularly the neutrophil count [3,5]. High-risk chemotherapeutic agents include doxorubicin, bleomycin, fluorouracil, and methotrexate, which are commonly integrated into pediatric treatment regimens [5,6].

The pathophysiology of OM unfolds in five sequential phases: initiation, signaling, amplification, ulceration, and healing. The initial insult involves the generation of reactive oxygen species (ROS), which disrupt epithelial integrity and activate the nuclear factor kappa B (NF-κB) pathway. This activation promotes the release of pro-inflammatory cytokines, aggravating mucosal injury and contributing to ulcer development. Subsequent microbial colonization of the damaged tissue further intensifies the local inflammatory response, perpetuating the cycle of mucosal breakdown and delayed recovery [3].

The objective of this study is to evaluate chemotherapy-induced mucositis in pediatric patients at the Pediatric Oncology Unit of Mohammed VI University Hospital in Oujda, and focus on its clinical presentation, progression, associated risk factors, and the effectiveness of therapeutic interventions used for its management.

## Materials and methods

This retrospective study was conducted at the Pediatric Oncology Unit of Mohammed VI University Hospital in Oujda, Morocco, from July 2022 to August 2023. The study received approval from the Institutional Ethics Committee of Mohammed First University, Oujda, Morocco, and written informed consent was obtained from the parents or legal guardians of all the participating children.

Inclusion criteria

Patients (between 3 and 16 years of age) with a confirmed diagnosis of a malignant tumor (solid or hematological), had undergone treatment with at least one cycle of chemotherapy at the Pediatric Oncology Unit during the study period, and had their complete medical and therapeutic records available, were included in the study.

Cases were included only when mucosal lesions appeared after the initiation of chemotherapy, in the absence of other identifiable causes such as viral infections, poor oral hygiene, or pre-existing mucosal conditions.

Exclusion criteria

Patients treated exclusively with radiotherapy or surgery without chemotherapy, those with incomplete or missing clinical data, those transferred from or to another institution before receiving full treatment at our center, and those who refused or withdrew their informed consent were excluded from the study.

Grading of mucositis

The severity of mucositis was classified according to the World Health Organization (WHO) oral toxicity scale [7]:

Grade 0: No mucositis

Grade 1: Erythema and soreness

Grade 2: Ulcers, able to eat solids

Grade 3: Ulcers, requires a liquid diet

Grade 4: Ulcers, alimentation not possible

A questionnaire was used to identify the occurrence of mucositis in patients undergoing chemotherapy. Data were retrieved from patients’ medical records. An Excel database (Microsoft Corp., Redmond, WA, US) was created to compile anamnesis, clinical findings, laboratory results, imaging data, and therapeutic information.

Primary and secondary outcomes

The primary outcome was the incidence and clinical profile of chemotherapy-related mucositis in pediatric patients with cancer during the study period.

Secondary outcomes included the identification of risk factors associated with the occurrence and severity of mucositis, as well as the assessment of its progression following appropriate management.

## Results

Among the 250 pediatric patients treated with chemotherapy during the study period, 145 (58%) developed chemotherapy-induced mucositis and were included in the analysis. These cases were recorded at the Pediatric Oncology Unit of Mohammed VI University Hospital in Oujda, Morocco. The age of the patients varied from three to 16 years, indicating a broad range. Male patients were predominant, accounting for 66% of the total cases. In addition, 51% of the patients had a previous history of mucositis, highlighting the recurrent nature of this complication (Table 1).

**Table 1:** Gender distribution and history of mucositis among pediatric patients with chemotherapy-induced mucositis

Gender	Percentage	Number of cases	History of mucositis	Without a history of mucositis
Male	66%	96	49 (51%)	47 (49%)
Female	34%	49	25 (51%)	24 (49%)

**Figure 1:**
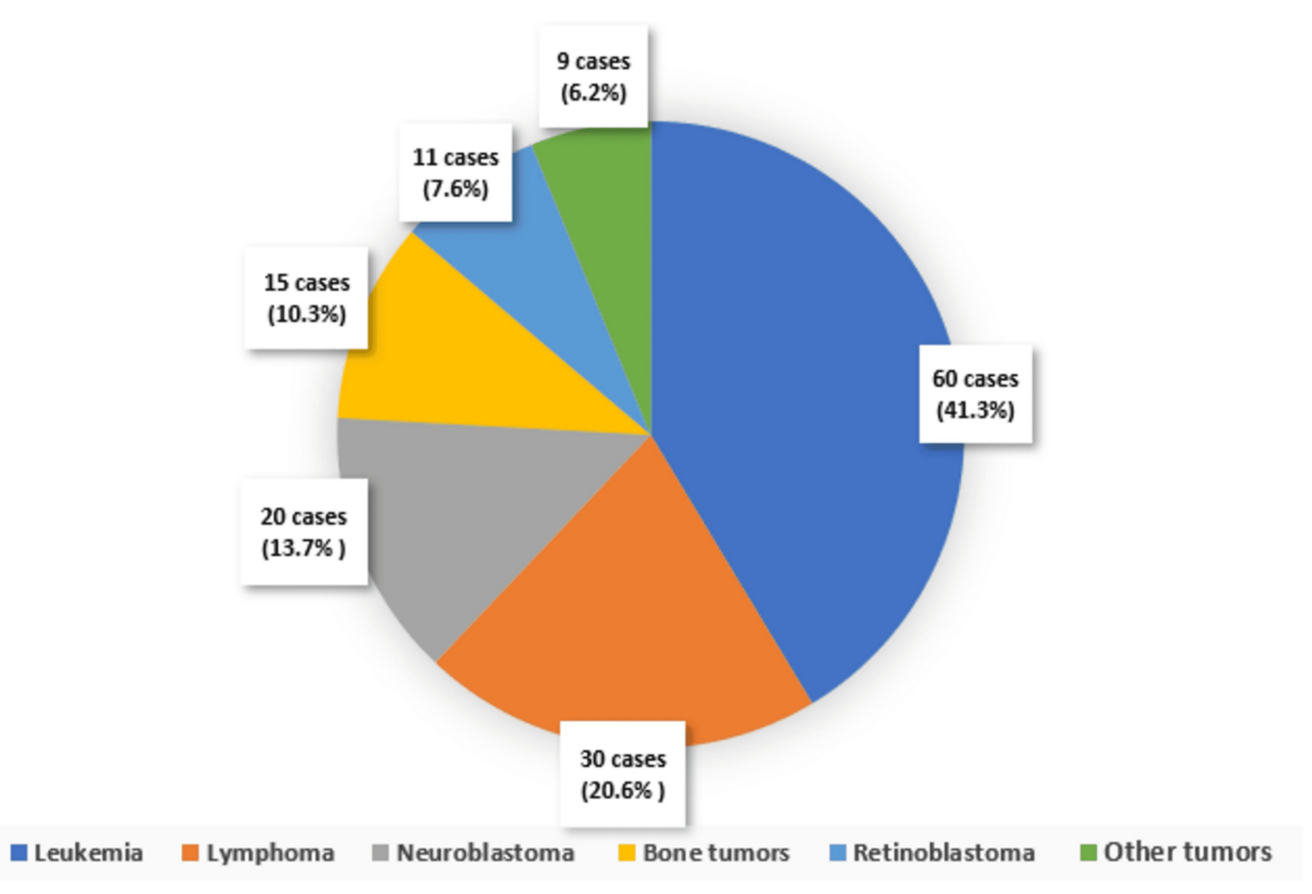
Distribution of cancer types among pediatric patients with chemotherapy-induced mucositis (n=145)

Leukemia was the most frequently observed malignancy, accounting for 41.3% of the cases, followed by lymphoma (20.6%), neuroblastoma (13.7%), bone tumor (10.3%), retinoblastoma (7.6%) and other tumors (6.2%). Leukemia, which often requires intensive chemotherapy regimens, was particularly associated with severe mucositis cases (Figure 1).

Regarding the clinical manifestations, pain emerged as a primary symptom, with the visual analog scale (VAS) for pain ranging from four to nine out of a score of 10, indicating that the pain was often intense, particularly in severe cases of mucositis. In some instances, opioid analgesics were required, highlighting the impact and severity of this complication. A refusal to eat and anorexia were nearly constant symptoms among these patients, exacerbating their nutritional status and potentially contributing to nutrient deficiencies, delayed healing, and worsening side effects of the chemotherapy. Diarrhea was noted in 38% of cases (n=55). Anorexia may be attributed to the oral pain and feeding difficulties, as well as the psychological stress or adverse effects of the chemotherapy. In addition, 66% of the patients exhibited significant signs of anxiety (Table 2).

**Table 2:** Prevalence of symptoms in patients with mucositis (n=145)

Symptom	Percentage (%)	Number of cases (n)
Pain	97%	141
Fever	75%	109
Anxiety	66%	96
Anorexia	95%	138
Diarrhea	38%	55
Dysphagia	45%	65

Based on the WHO oral toxicity scale, approximately 50% (n=72) of the patients had grade 1 mucositis, characterized by moderate symptoms such as oral pain and difficulty eating, without severe deterioration of the general health, 30% (n=44) were classified as grade 2, and 20% (n=29) as grade 3. 

Notable abnormalities were detected in the blood parameters of the patients. Neutropenia was present in 80% of the cases, indicating a marked suppression of the immune function. Furthermore, 63% (n=73) of the patients who had neutropenia also presented with grade 2 or 3 mucositis, highlighting a significant association between neutropenic status and the severity of mucositis. Lymphopenia, present in 33% of the patients, suggested increased immunosuppression and dysfunction of the cellular immune response. Additionally, C-reactive protein (CRP) was elevated in 79% of the cases, serving as a biomarker of systemic inflammation, consistent with the inflammatory processes associated with mucositis.

Therapeutic management of chemotherapy-induced OM at the Pediatric Oncology Unit of Mohammed VI University Hospital is based on a standardized multidisciplinary protocol aimed at minimizing complications and improving patient comfort. Preventive care includes systematic dental assessment prior to chemotherapy, maintenance of rigorous oral hygiene with twice-daily brushing using a soft-bristled toothbrush, and regular rinsing with a bicarbonate solution (composed of one-fourth teaspoon each of sodium bicarbonate and salt dissolved in 250 mL of lukewarm water, to be used four to six times per day). Oral cryotherapy, involving the administration of ice, is utilized as a prophylactic intervention in specific chemotherapy protocols, such as those incorporating 5-fluorouracil or melphalan, to reduce the incidence and severity of mucositis.

For symptom management, treatment is tailored to the severity of mucositis. Mild to moderate cases may be relieved with paracetamol (15 mg/kg, four times daily) and topical antifungal agents such as miconazole. For more severe forms, topical analgesics like 2% viscous lidocaine (5 mL, three to four times daily), especially before meals, are used to ease oral discomfort. In cases of significant pain, systemic analgesia is required, involving oral morphine (0.2-0.3 mg/kg every four hours) or intravenous administration (0.05-0.1 mg/kg every two to four hours). In our study, 30% of the patients (n=44) required injectable morphine for effective pain control.

Topical treatments like sucralfate suspension (1 g four times/day) help protect ulcerated mucosa. Antifungal agents such as nystatin (100,000 IU/mL suspension) are used in suspected oral candidiasis, with fluconazole (6 mg/kg/day orally or IV) as second-line therapy for severe or unresponsive cases. Empiric antibiotic therapy, typically with amoxicillin-clavulanate (80-100 mg/kg/day), is initiated in cases of suspected bacterial superinfection, especially in the presence of neutropenia or advanced mucositis.

Nutritional support varies by severity: patients with grades 1-2 mucositis are advised to consume a soft, bland diet, while those with grade 3 may require nasogastric feeding or total parenteral nutrition. Medical-grade honey is occasionally used as an adjunct for its anti-inflammatory and healing properties.

All our patients received personalized psychological support aimed at emotionally supporting them throughout their treatment. This included regular sessions with specialized psychologists to help them cope with pain, anxiety, and the emotional challenges associated with chemotherapy and its side effects, particularly mucositis.

The development of mucositis caused temporary interruptions in chemotherapy for 15% cases, resulting in treatment delays and requiring modifications to the therapeutic protocols. In our cohort, 95% of the patients with chemotherapy-induced mucositis experienced favorable outcomes when given appropriate care.

## Discussion

Mucositis is an acute inflammation of the mucosal lining, which can affect the entire digestive tract. This encompasses OM, which affects the oral cavity and upper aerodigestive tract, and gastrointestinal mucositis, primarily involving the small intestine, and proctitis, which occurs when the rectal mucosa is affected. Clinically, mucositis can manifest as inflammation, erythema, ulcerations, bleeding, or swelling of the mucosal surfaces. Oropharyngeal involvement is the most common manifestation. In cases of gastrointestinal involvement, diarrhea is the primary symptom. However, other clinical signs, including nausea, vomiting, and abdominal or rectal pain, have also been reported. The symptoms can range from a few days to several weeks in duration [8].

OM is a common adverse effect of chemotherapy, which can be influenced by several variables, including the kind of cancer, the chemotherapy regimen, the type of chemotherapeutic agents administered, the patient's characteristics, oral hygiene status, and neutrophil count [9]. The reported incidence rates vary widely, ranging from 16.7% to 91.5% [3]. In our study, mucositis was observed in 58% (n=145) of the 250 pediatric patients undergoing chemotherapy with consistent follow-up, corroborating its high prevalence in this population. 

OM results from interactions between oral mucosal cells, pro-inflammatory cytokines, and local environmental factors [10]. Its severity varies significantly among pediatric patients receiving chemotherapy. While some develop severe mucosal lesions, others may exhibit minimal or no symptoms. In a prospective study conducted at Donka University Hospital, it was found that among 69 children hospitalized for cancer, 48 developed chemotherapy-induced gastrointestinal complications, with mucositis observed in 47.92% of the patients [11]. Similarly, mucositis was frequently observed in our series. In contrast, a case series by Garrocho-Rangel et al. [12] reported that none of the 11 children treated with methotrexate during a 14-day observation period developed severe OM.

The diagnosis of mucositis is primarily based on clinical evaluation, as it presents with recognizable signs and symptoms. In chemotherapy-induced cases, the condition predominantly involves the mobile areas of the oral mucosa, with the dorsal tongue, hard palate, and gingiva less commonly affected [13]. Commonly reported symptoms include oral pain, nausea, vomiting, reduced appetite resulting in weight loss, fatigue, as well as disturbances in sleep and mood [14]. In our study, pain emerged as a primary symptom.

Acute lymphoblastic leukemia and retinoblastoma were the most common diagnoses, each accounting for 25% of the cases [11]. In our study, leukemia and lymphoma were the most frequent diagnoses. Mucositis is frequently assessed using well-established classification systems, such as those developed by the WHO and the National Cancer Institute’s Common Terminology Criteria for Adverse Events (NCI-CTCAE) [15]. 

Some hematological parameters such as neutrophil, platelet, and creatinine counts are possible risk factors for OM [4]. As the neutrophil count decreases, the severity of OM increases in pediatric oncology patients who have received chemotherapy [16]. Neutropenic children are 7.5 times more likely to develop OM [17]. In our study, neutropenia was present in 80% of the cases, indicating a marked suppression of the immune function. Furthermore, 63% (n=73) of the patients who had neutropenia also presented with grade 2 or 3 mucositis, highlighting a significant association between neutropenic status and the severity of mucositis. However, it is not possible to establish any association of hematological parameters with the occurrence and severity of OM in this study.

In immunocompromised children, mucosal injury also increases susceptibility to opportunistic infections of fungal, viral, or bacterial origin. Infections of this nature can progress to systemic involvement, necessitating hospitalization and intensive medical management, which may incur significant healthcare costs [14]. In more severe cases, these complications may require adjustments or temporary suspension of anticancer therapies, potentially compromising the overall prognosis [14]. In our study, the development of mucositis caused temporary interruptions in chemotherapy for 15% of cases. 

Preventive strategies for mucositis involve oral hygiene management through rigorous oral care and the use of mild mouthwashes such as saline or sodium bicarbonate solutions. These basic measures are widely recognized in the literature as essential for preserving the integrity of the oral mucosa. For instance, the study by Elad et al. [18] emphasizes the importance of these basic interventions in preventing mucosal lesions in chemotherapy patients. Cryotherapy, which involves the use of ice during the administration of certain chemotherapeutic agents (notably 5-fluorouracil), has also demonstrated efficacy in reducing the incidence of mucositis [7].

The guidelines of Elad et al. [18], within the framework of Multinational Association of Supportive Care in Cancer/International Society of Oral Oncology (MASCC/ISOO) recommendations, stress the importance of using potent analgesics, including morphine, for managing severe forms of mucositis. In our study, 30% (n=44) of the patients required injectable morphine for pain management. This finding underscores the need for aggressive pain control in severe cases of mucositis, in accordance with international recommendations. Moreover, Elad et al. [18] also showed that early initiation of empirical antibiotic therapy in patients with grade 3-4 mucositis associated with neutropenic fever significantly reduced hospitalization duration and infectious complications. These findings highlight the importance of integrating either prophylactic antibiotic strategies or curative treatment based on the patient's clinical status and symptom severity.

Lastly, appropriate nutritional support is crucial to maintain adequate hydration and caloric intake. Pre-treatment dental consultations are also recommended, as advocated by the French National Cancer Institute (INCa) guidelines, to minimize the risk of oral complications and reduce the severity of mucositis [19]. 

In conclusion, this study highlights the critical role of a preventive and multidisciplinary approach in managing chemotherapy-induced mucositis in pediatric oncology patients. Effective management should address the clinical, biological, and psychological aspects of the condition to improve the overall quality of life for young patients, minimize pain, anxiety, and treatment interruptions, and enhance clinical outcomes. Despite the valuable insights gained from this study, certain limitations, such as the single-institution design and the relatively homogenous sample size, may limit the generalizability of the findings to broader pediatric oncology populations and healthcare settings. In addition, our study did not include a control group, which limits our ability to compare the effects of the intervention or exposure to a baseline. Future research incorporating a control or comparison group will be important for evaluating the effectiveness and causal impact of the intervention. 

## Conclusions

Mucositis is a frequent complication of oncological treatments, often presenting with severe pain that profoundly affects the patient's health and quality of life. In addition, this condition also facilitates the development of various infections. Its management remains a major concern for both clinicians and patients. It can compromise functional outcomes and overall prognosis, particularly when cancer treatment must be discontinued. Various therapeutic options are available, and effective pain management requires a multimodal approach, incorporating both local and systemic therapies, including opioids, non-pharmacological interventions, and preventive measures. Early prevention and management are critical factors for the optimal management of chemotherapy-induced mucositis.
